# Identifying thresholds in air exposure, water temperature and fish size that determine reflex impairment in brook trout exposed to catch-and-release angling

**DOI:** 10.1093/conphys/coac070

**Published:** 2022-12-16

**Authors:** Jacob W Brownscombe, Taylor D Ward, Liane Nowell, Robert J Lennox, Jacqueline M Chapman, Andy J Danylchuk, Steven J Cooke

**Affiliations:** Great Lakes Laboratory for Fisheries and Aquatic Sciences, Fisheries and Oceans Canada, 867 Lakeshore Road, Burlington, Ontario, L7S 1A1, Canada; Fish Ecology and Conservation Physiology Laboratory, Department of Biology and Institute of Environmental and Interdisciplinary Science, Carleton University, 1125 Colonel By Dr., Ottawa, Ontario, K1S 5B6, Canada; Fish Ecology and Conservation Physiology Laboratory, Department of Biology and Institute of Environmental and Interdisciplinary Science, Carleton University, 1125 Colonel By Dr., Ottawa, Ontario, K1S 5B6, Canada; Kenauk Institute, 1000 Chemin Kenauk, Montebello, Quebec, J0V 1L0, Canada; NINA – Norwegian Institute for Nature Research, Høgskoleringen 9, Trondheim, 7034, Norway; Fish Ecology and Conservation Physiology Laboratory, Department of Biology and Institute of Environmental and Interdisciplinary Science, Carleton University, 1125 Colonel By Dr., Ottawa, Ontario, K1S 5B6, Canada; Department of Environmental Conservation, University of Massachusetts Amherst, 160 Holdsworth Way, Amherst, MA, 01003, USA; Fish Ecology and Conservation Physiology Laboratory, Department of Biology and Institute of Environmental and Interdisciplinary Science, Carleton University, 1125 Colonel By Dr., Ottawa, Ontario, K1S 5B6, Canada

**Keywords:** water temperature, synergistic effects, recreational fisheries, post-exercise recovery, Air exposure

## Abstract

Understanding the factors that contribute to fish impairment and survival from angling events is essential to guide best angling practices for catch-and-release (C&R) recreational fisheries. Complex interactions often exist between angler behaviour, environmental conditions, and fish characteristics that ultimately determine biological outcomes for fish. Yet, few studies focus on identifying biologically relevant thresholds. We therefore examined the effects of water temperature, air exposure and fish size on reflex impairment and mortality in brook trout *Salvelinus fontinalis* exposed to experimental and simulated angling stressors (*n* = 337). Using conditional inference trees, we identified interactions among these factors as well as threshold values within them that determine brook trout reflex impairment as an indicator of whole animal stress. Specifically, longer air exposure times (>30 sec) and warmer temperatures (>19.5°C) had a synergistic effect leading to higher reflex impairment scores. Further, larger fish (>328 mm) were more sensitive to air exposure durations >10 sec. Of the reflex impairment measures, loss of equilibrium and time to regain equilibrium were strongly and moderately associated with brook trout mortality (18–24 h monitoring), although mortality rates were generally low (6%). These findings support previous research that has established strong links between these reflex impairment measures and fish health outcomes in other species. They also highlight the important interactions among air exposure duration, water temperature and fish size that determine impairment in brook trout, providing specific thresholds to guide best angling practices for C&R fisheries. This approach may be widely applicable to generate similar thresholds that can be encouraged by regulators and adopted by anglers for other common C&R fishes.

## Introduction

Recreational angling is a popular activity around the globe, with many anglers employing catch-and-release (C&R) practices, whereby angled fish are subsequently released to comply with harvest regulations or because of angler conservation ethic (Cooke and Cowx, 2004; Arlinghaus *et al.,* 2007). The efficacy of C&R as a management and conservation strategy is predicated on the assumption that fish released incur minimal injury, physiological disturbance and behavioural impairment and is able to recover with negligible fitness impairments (Cooke and Schramm, 2007). However, in many cases fish that are caught and released are subject to perturbations to homeostasis (e.g., physical damage or physiological stress), which can result in a variety of behavioural consequences, the ecological relevance of which remains poorly understood (Cooke *et al.,* 2013). As such, a growing body of literature is focused on parsing the mechanisms that influence the fate of released fish, with the goal of determining biologically relevant endpoints to fish welfare and fitness (Cooke and Suski, 2005; Brownscombe *et al.,* 2017).

Fish are exposed to potential stressors at each stage of the C&R process, meaning that angler decisions throughout a capture event are crucial to fish welfare (Cook *et al.,* 2015; Brownscombe *et al.,* 2017). Decisions about angling technique (e.g. passive vs. active methods), gear type (e.g. hook size and shape, barbed vs. barbless hooks, landing net type), as well as handling technique are focal points of management strategies and voluntary actions in many recreational fisheries. Angler behaviours may also interact with, or be mediated by, environmental conditions (e.g. water temperature, capture depth) that can impose greater physiological stress on angled fish. Therefore, developing species- and context-specific angler guidelines (i.e. related to specific water bodies, seasons or practices) has become a priority for C&R science because of its potential to improve fish welfare (Cooke and Suski, 2005).

Water temperature is a well-resolved factor controlling ectotherm metabolism and performance and is implicated in mediating physiological processes, such as growth, and response to exhaustive exercise and perceived stressors (Fry, 1947). Thermal conditions of aquatic environments are expected to not only influence the availability of dissolved oxygen directly, but also limit the ability of fish to cope with stressors such as oxygen depletion from exhaustive exercise (anaerobic metabolism). Exhaustive exercise and air exposure can have synergistic effects on fish impairment and recovery (Ferguson and Tufts, 1992), and water temperature can further modulate this interaction (Gingerich *et al.,* 2007; Gale *et al.,* 2011). Because water temperature determines many biological limits, evaluating its effect on C&R outcomes can establish valuable thresholds (i.e. specific values within which negative fish health outcomes occur significantly more often) for this fundamental fisheries management variable (e.g. temperature-based fisheries closures). Larger-bodied fishes also have higher absolute metabolic needs (Clarke and Johnston, 1999), and greater swimming capacity, often leading to greater physiological stress responses and reduced survival relative to smaller-bodied fish of the same species (Cooke and Suski, 2005). Despite this body of work, thresholds for these factors are notably lacking, which makes it difficult to provide anglers, regulators and managers with science-based guidance for C&R.

Given that there is evidence that interactions among angling practices, environmental conditions and fish characteristics ultimately determine the impacts of C&R angling events on fish health and survival, the purpose of this study was to explore interactions among key factors that determine biological outcomes for fish from C&R angling including air exposure duration, water temperature and fish size. This study focused on brook trout (*Salvelinus fontinalis*), which are a popular recreationally targeted species (Karas, 2015) that is generally regarded as being rather sensitive to fisheries interactions (Schreer *et al.,* 2005; Chadwick *et al.,* 2015). We focused on reflex impairment metrics as responses due to their well-established association with fish health and fitness outcomes from stressors including C&R (Davis, 2010; Raby *et al.,* 2012; Brownscombe *et al.,* 2017). In doing so, we aimed to identify key thresholds in these factors that can contribute to further development of C&R best practices for brook trout. Biological thresholds are of great utility but are rarely incorporated into resource management (Mavrommati *et al.,* 2016) aside from the context of environmental toxicology (Aldridge, 1986).

## Materials and methods

### Experimental design

This study consisted of two experimental procedures that were conducted concurrently, which will hereafter be referred to as ‘Experimental angling’ and ‘Simulated angling’. Research was conducted at Kenauk Nature, a private wilderness reserve near Montebello, Quebec, Canada, with lakes stocked with brook trout (45.712538, −74.888264). Experiments occurred on four small natural lakes, including Collins (*n* = 211, surface area = 0.12 km^2^, mean depth = 9 m), Jackson (*n* = 15, 0.05 km^2^, 1.5 m), Moose (*n* = 44, 0.05 km^2^, 3 m) and Mountain (*n* = 67, 0.03 km^2^, 3 m). Brook trout in these lakes originate from the same hatchery, and stocking data indicates similar densities among lakes (not reported). Sampling occurred from 11 June to 8 October 2015. Water temperatures were measured at 1 m subsurface (Handy Polaris Portable Dissolved Oxygen Meter, OxyGuard International AS, Farum, Denmark) and varied from 12.2–23.3°C during the study period.

#### Experimental angling

In the experimental angling experiment, fish were captured directly from a wild setting, with air exposure treatments applied at the time of capture, and is distinguished from the simulated angling method, which used previously retained fish and employed an angling simulation prior to air exposure treatments (Simulated Angling Section below). In experimental angling, fish were captured by rod-and-reel angling using fast-action, light spinning rods with assorted in-line spinner lures and barbed single-hooks by trolling or casting in the top 5 m of the water column. The angling protocol followed that of a previous study at this location and was selected as these gear types are expected to minimize reflex impairment of brook trout in these lakes (Kerr *et al.,* 2016). Angled fish were landed in a rubberized mesh net, de-hooked while in water, held out of water in the landing net for a controlled duration, returned to a cooler filled with lake water at ambient surface temperature and dissolved oxygen concentration, immediately evaluated for reflex impairment (see Section 2.1.3) and transported to an *in situ*, floating mesh holding pen (2 × 2 m wide, 3 m deep).

During the angling event, several parameters were recorded including the duration of the angling event from the instance of hooking until the fish was landed (‘fight time’ in seconds), the time taken for hook removal (‘handling time’ in seconds), as well as the hooking location and the presence of bleeding. Fish were randomly assigned to treatment categories of 0, 10, 30, 60 or 90 sec of air exposure. These treatment groups were selected because they span the realistic duration of the fish landing and admiration period following angling ranging from short to prolonged periods; Roth *et al.,* 2018), and correspond to previously investigated air exposure durations in salmonids (Ferguson and Tufts, 1992). Each individual was measured (total length, mm), marked with a uniquely coded plastic tag inserted using dorsal puncture and retained in the net pen overnight to assess short-term (18–24 h) survivorship.

#### Simulated angling

The simulated angling experiment involved exposing fish to an angling simulation event and subsequent air exposure treatments to fish that were previously retained overnight, and as such were subject to different pre-treatment conditions than fish in the experimental angling procedure. Fish in the simulated angling experiment were captured via angling in the same manner as the experimental angling method and were held for 18–24 h in net pens to assess short-term survivorship for a concurrent investigation of fish response to different landing net types (Lizée *et al.,* 2018). All fish in the simulated angling group were assessed as ‘not impaired’ (see Section 2.1.3) prior to the angling simulation process based on reflex responses. The standardized protocol for fish in the simulated angling group is as follows: fish were netted individually from within the net pen, fish received an ‘angling simulation’ event consisting of a 20-sec burst swimming challenge while being held in a landing net at the air–water interface, fish were then held out of water in the landing net for a controlled duration, then transferred to a cooler with lake sourced water, immediately evaluated for reflex impairment (see Section 2.1.3) and released. Simulated angling fish were not retained for observation of short-term mortality.

#### Reflex tests

Reflex impairment is an increasingly popular approach for evaluating fish stress and mortality probability, as reflex impairment reflects the cumulative effect of different stressors and is applicable across contexts and fish sizes, ages and motivational states (Davis, 2010). Using reflex impairment as an indicator of fish health provides a simple and informative tool for anglers or managers to assess relative levels of impairment following angling. The procedure provides an intuitive, low-cost and non-invasive measure of fish condition and is easily operationalized in field conditions (Cooke *et al.,* 2013). To make inferences about the fate of individual fish based strictly on reflex tests requires detailed experimental validation of mortality events (Davis, 2010), which our study does not purport to attempt. Here, we use RAMP score as a metric of relative impairment among fish, and verify the validity of our approach by monitoring short-term (<24 h) mortality rates.

In this study RAMP scores were determined using a set of reflex tests previously shown by Raby et al. (2012) to correlate with delayed mortality in coho salmon (*Oncorhynchus kisutch*), including *tail grab*, *body flex* and *orientation* tests. The *tail grab* test evaluates a fish’s startle response, and corresponds to a forward movement in response to an abrupt grabbing motion directed towards the fish’s tail; *body flex* similarly evaluates a fish’s innate escape reflex and tensing of the body musculature in response to restraint around the body; *orientation* assesses the fish’s righting reflex described as the ability to maintain normal upright orientation, as in a free-swimming fish, after being rolled into an inverted position. Fish that responded positively to a reflex test were assigned a value of 0, while a negative response was scored as 1, meaning a fish failing to exhibit any positive reflex responses is scored as 3. For fish that were unable to regain orientation after 5 sec, we measured the time needed to regain equilibrium (‘Time to Equilibrium’ in seconds).

### Statistical analyses

All analyses were conducted using R v1.2.5019 (R Core Team, 2019). Brook trout RAMP scores were compared between experimental and simulated angling experiments using ordinal logistic regression fit with the polr function in the MASS R package (Venables and Ripley, 2002), with RAMP score (0–3) treated as an ordered factor response, experiment (experimental, simulated) and air exposure treatment (0, 10, 30, 60, 90) as categorical factors and water temperature (°C) as a numeric predictor, as well as interactions between experiment and treatment and experiment and water temperature. Model structure was assessed by comparing models with and without interactions with Akaike information criterion (AIC). Tukey’s HSD *post hoc* test was used to assess significant differences within each RAMP value between experimental types using the emmeans R package (Lenth, 2020). The slopes between water temperature and RAMP score were compared between the two experiments with a Tukey’s HSD using the ‘emtrends’ function in emmeans. Relationships between RAMP metrics and brook trout mortality were assessed with a series of binomial generalized linear models with mortality (0 or 1) as the response and RAMP score, loss of equilibrium and time to regain equilibrium as predictors. Separate models were fit with each predictor due to collinearity. For all statistics, outputs were interpreted as no evidence (*P* > 0.1), weak evidence (*P* = 0.05–0.1), moderate evidence (*P* = 0.01–0.05) or strong evidence (*P* < 0.01).

Despite differences in RAMP scores between experimental and simulated angling datasets (reported in Results), the full dataset was used to explore thresholds in angling factors due to a need for relatively large and robust sample sizes. The full dataset was fit with a decision tree algorithm, conditional inference trees (CIT), to investigate biologically relevant thresholds of impairment. Decision tree algorithms use recursive partitioning to predict the response variable with the capacity to integrate hierarchical effects of large numbers of potential predictor variables (Breiman, 1984). These algorithms are also more robust to typical statistical assumptions such as normality, co-linearity, data distribution and missing values, making them valuable for exploring the effects of complex multivariate datasets. CIT use a criterion of statistical evidence to determine the number of splits to avoid biases of traditional approaches including overfitting and favouring variables with more levels (Hothorn *et al.,* 2006). CITs were fit to three response variables: RAMP score (categorical; 0 to 3), loss of equilibrium (categorical; 0 or 1) and time to regain equilibrium (continuous; seconds), with air exposure treatment (categorical; 0, 10, 30, 60, 90 sec), water temperature (continuous; 19.5 ± 3.5°C; mean ± SD; 12.0–23.3°C range) and fish total length (continuous; 320 ± 41.4 mm; mean ± SD; 240–468 mm range) as predictors. Fight time prior to landing was not included due to low variability in that measure. CITs were fit using the ctree function (Hothorn *et al.,* 2006) in the partykit R package (Hothorn and Zeileis, 2015). Default test statistics in the ctree function were used to determine moderate to strong support for data partitions.

## Results

**Figure 1:**
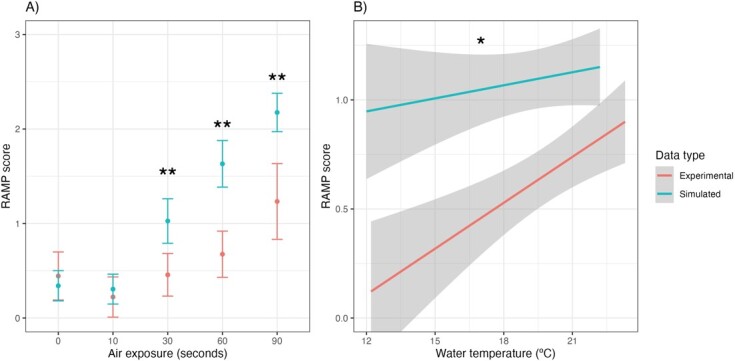
A) Relationship between air exposure period (seconds) and brook trout RAMP scores (0 to 3), and B) water temperature and RAMP scores from real and simulated angling experiments. Single * indicates moderate statistical evidence, double **indicates strong statistical evidence.

In total 337 fish were sampled, including experimental angling (*n* = 141) and simulated angling (*n* = 196). There was no evidence of a relationship between fish total length (320 ± 2 mm; mean ± SE) and water temperature (linear model; t_332_ = 1.08, *P* = 0.28), but there was weak evidence of a relationship between fish length and air exposure treatment (linear model; F_4,329_ = 2.20, *P* = 0.07), with 60 and 90 sec of air exposure having slightly higher mean fish sizes (Table 1). Comparing experimental vs. simulated angling experiments, there were important interactions between experiment and air exposure treatment ($\delta$AIC = 9.5), as well as experiment and water temperature ($\delta$AIC = 5.5) in predicting brook trout RAMP scores. There was strong evidence supporting differences among treatments in RAMP scores with 30 sec of air exposure (z = −3.903, *P* = 0.004), as well as 60 sec (*z* = −5.79, *P* < 0.001) and 90 sec (*z* = −4.71, *P* < 0.001), where RAMP scores were higher in the simulated angling experiment (Fig. 1A). There was also moderate evidence for differences in slopes between experiments in the relationship between water temperature and RAMP score (*z* = 2.30, *P* = 0.02; Fig. 1B). Fish in the simulated angling experiment experienced higher RAMP scores at colder temperatures, and fish in the experimental angling experiment exhibited a steeper increase in RAMP scores with temperature, with values nearly converging between treatments above 20°C (Fig. 1B).

**Table 1:** Summary statistics for brook trout experimental treatments (0–90 sec of air exposure) including overall sample size, experimental angling and simulated angling sample sizes and mean ± standard deviation (range) of water temperature and fish total length

Air treatment	Sample size	Experimental	Simulated	Water temperature (°C)	Total length (mm)
0	62	18	44	19.7 ± 3.1 (12.0–23.3)	315 ± 41 (245–424)
10	53	18	36	20.5 ± 2.6 (12.0–23.3)	316 ± 38 (255–390)
30	73	35	38	19.2 ± 3.7 (12.0–23.3)	312 ± 36 (240–380)
60	77	40	38	19.2 ± 3.8 (12.0–23.3)	326 ± 41 (247–430)
90	69	30	40	19.0 ± 3.7 (12.0–23.2)	329 ± 48 (255–468)

**Figure 2:**
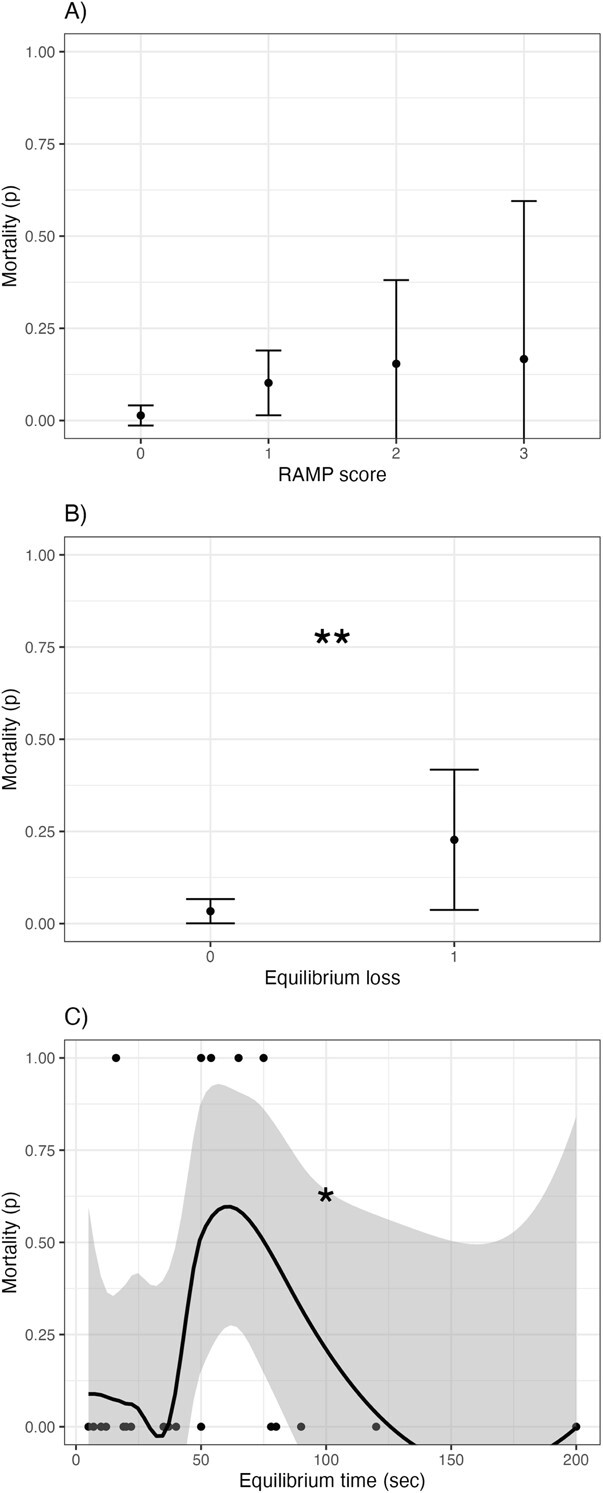
Relationships between brook trout A) RAMP score and mortality (probability), B) equilibrium loss and mortality, and C) time to equilibrium gain (seconds) and mortality. Single * indicates moderate statistical evidence, double ** indicates strong statistical evidence.

Fish mortality was measured only in the experimental angling dataset, within which brook trout mortality rates were 6% (9 of 141 individuals). RAMP metrics had varied relationships with brook trout mortality (Fig. 2). There was weak evidence of a relationship between RAMP score and brook trout mortality (ordered logistic regression, linear component; *z* = 1.86, *P* = 0.06; Fig. 2A). However, there was strong evidence of a positive relationship between equilibrium loss and mortality (*z* = 2.97, *P* = 0.003; Fig. 2B) and moderate evidence of a relationship between time to equilibrium gain and mortality (*z* = 2.07, *P* = 0.04; Fig. 2C). Mortality occurred more frequently at intermediate to long time to equilibrium gain values, although two fish with outlying long time to equilibrium gain values survived.

**Figure 3:**
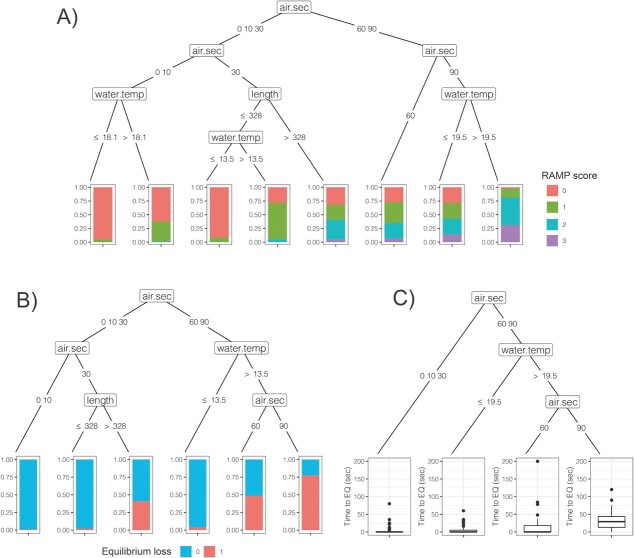
Conditional inference trees fit to brook trout A) RAMP scores, B) equilibrium loss, and C)time to equilibrium gain. Nodes indicate partitions in the data with moderate to strong statistical support (p<0.05), with values on the branches indicating the threshold cutoff values within the node above. air.sec=seconds of air exposure, water.temp=water temperature, length=fish total length (mm).

Examining the entire dataset with experimental angling and simulated angling combined, CIT models identified numerous partitions with moderate or strong support in predictor variables of brook trout RAMP score (Fig. 3A), equilibrium loss (Fig. 3B) and time to regain equilibrium (Fig. 3C). Air exposure periods of 60–90 sec resulted in greater RAMP scores compared to 0–30 sec, and within the 90 sec air treatment, there was also strong evidence for higher RAMP scores at >19.5°C (Fig. 3A). Brook trout equilibrium loss was also higher at 60–90 sec of air exposure, within which, there was strong support for greater equilibrium loss at >13.5°C (Fig. 3B). Equilibrium loss was very rare at 0–10 sec of air exposure, while at 30 sec air exposure, fish >328 mm in length exhibited greater rates of equilibrium loss (Fig. 3B). For those fish that lost equilibrium, 60–90 sec of air exposure resulted in longer times to regain equilibrium, especially at >19.5°C, within which, 90 sec of air exposure resulted in greater times to equilibrium gain than 60 sec (Fig. 3C).

## Discussion

This study aimed to characterize the complex interactions between some of the most influential factors related to biological outcomes for fish exposed C&R events including angler behaviour (air exposure duration), water temperature and fish size. There were clear interactive effects of these factors on brook trout impairment, with thresholds identified in air exposure duration (>30 sec). Within these longer air exposure times, water temperature had a synergistic effect, exacerbating reflex impairment and time to regain equilibrium at >19.5°C, and >13.5°C for equilibrium loss such that trout were more likely to lose equilibrium beyond 13.5°C and took longer to recover beyond 19.5°C. Further, larger fish (>328 mm) were more vulnerable to equilibrium loss at more minimal periods (>10 sec) of air exposure, indicating greater sensitivity to this factor. Notably, these larger fish would be the most likely group to be air exposed by anglers seeking to take pictures or otherwise admire their catch. Although brook trout mortality risk factors were not assessed comprehensively, with only 9/141 fish (6%) experiencing mortality, there was weak evidence of a relationship with RAMP score, strong evidence with equilibrium loss and moderate evidence with time to equilibrium gain and mortality. There is a large body of empirical evidence to support that there are strong relationships between RAMP metrics, especially equilibrium loss, with fish mortality and other fitness-related outcomes (e.g. Davis, 2010; Raby *et al.,* 2012; Brownscombe *et al.,* 2013). Therefore, the thresholds described here may serve as benchmarks for guiding brook trout C&R best practices. The capacity to generate these benchmarks was enabled by a combination of comprehensive data collection (*n* = 337), as well as the application of a novel analytical technique (i.e. CIT) sourced from the field of machine learning.

Comparing experiments, brook trout reflex impairment was greater from the simulated angling experiment, where fish were captured by rapid angling, held in near-surface net pens *in situ* for 18–24 h and then exposed to simulated angling stressors. In experimental angling, brook trout were captured with more typical real-world angling practices and assessed immediately. Importantly, water temperatures below 1 m sub-surface were not measured. The thermal biology of brook trout (cool-water species, discussed extensively below) is such that they may have been residing in colder water than our surface measurements prior to capture, although they were mainly captured near surface and their vertical positions prior to capture are not known. Assessing the full thermal profiles of the lakes would have improved study design. We can, however, assess how it may have impacted brook trout reflex impairment responses by comparing experiments. Brook trout reflex impairment was higher in simulated angling than experimental angling at colder water temperatures (when thermal profiles of lakes are likely to be more consistent), but responses were similar between experiments at warmer temperatures (when there may have been a thermal gradient). This may indicate that potential exposure to a thermal gradient during experimental angling (i) caused higher fish stress, at the same level as simulated angling, or (ii) was not a major factor, as outcomes were similar. The effects of depth-based variability in temperature on fish health outcomes is worthy of further study in fishes that occupy relatively cold water in systems with a thermocline and where small changes in depth (e.g. a few meteres) could lead to large swings in water temperature. In small- to medium-sized riverine systems that are well-mixed thermally, temperatures may be homogenous within a reach such that body temperature can be inferred with reasonable confidence from surface water temperature (e.g. Meka and McCormick, 2005). Yet, that does not work in most deeper or more lentic systems where temperature varies with depth during seasonal stratification. In those situations, future studies could examine vertical temperature profiles of an aquatic system as well as fish depth use at capture (perhaps using underwater cameras or hydroacoustics/sonar) to better assess the role of temperature variation in fish stress responses. Another study approach could be to measure fish body temperatures directly upon capture to gain accurate measures of body temperature relevant to stress responses. Recent work involving the thermal imaging of sharks has proved useful in C&R studies (Wosnick *et al.,* 2018). Moreover, the fact that our experiment that simulated angling resulted in greater reflex impairment responses than experimental angling means that the thresholds developed here using both approaches are likely conservative for real angling scenarios.

It is not surprising that air exposure was a dominant factor determining brook trout reflex impairment, because it is widely recognized from previous studies as a key determinant of reflex impairment, physiological stress, and mortality (Cooke and Suski, 2005; Cook *et al.,* 2015). However, fish species exhibit a range of tolerance levels to air exposure, likely related to their metabolic needs and capacity to use anaerobic pathways (Brownscombe *et al.,* 2017a; Ern, 2019). Intuitively, fish species with strong oxygen dependence in their metabolic and stress responses, such as brook trout, are likely more sensitive to oxygen deprivation relative to species that can readily employ anaerobic metabolic pathways. This study also highlights that air exposure thresholds cannot be considered independent of other factors, predominantly water temperature, as well as fish size. Specifically, brook trout were more robust to up to 90 sec of air exposure when water temperatures were <13.5°C, rarely losing equilibrium within this colder temperature range. Independent of temperature, brook trout rarely lost equilibrium with ≤30 sec of air exposure, but larger fish (>328 mm) lost equilibrium substantially more often at >10 sec of exposure. These interactions are largely specific to species. For example, largemouth bass can frequently lose equilibrium after just 30 sec of air exposure at >25°C, but tolerate over 10 min of air exposure at <12°C (Thompson *et al.,* 2008; Brownscombe *et al.,* 2014; Brownscombe *et al.,* 2017b). Despite inter-species variability, our findings and those of other studies support the ‘10-sec rule’ as a relatively simple and safe guideline for air exposure limits, allowing sufficient time for angler admiration without causing significant harm during C&R practices (Cook *et al.,* 2015). This precautionary approach is particularly important at warm water temperatures relative to species’ physiology (i.e. >19°C for brook trout) and relatively large individuals (>328 mm in brook trout).

The synergistic effects of air exposure with water temperature and fish size are consistent with fundamental knowledge of biological processes. Fish metabolic rate (and thus, oxygen needs) increase predictably with body mass and water temperature (Clarke and Johnston, 1999). Hence, although the specific threshold values identified here in water temperature and body length serve as useful C&R benchmarks, the synergistic effects of air exposure likely increase on a continuum with these factors. Larger fish are commonly more sensitive to angling stressors, likely due to a combination of increased metabolic needs, as well as strong swimming abilities and increased capacity to fight against anglers, resulting in longer fight times (Meka and McCormick, 2005). However, it has been suggested that small–medium-bodied fishes angled with species-appropriate fishing gear rarely generate fight times beyond seconds to a few minutes, and so fight time is less of a factor than it is for large-bodied species that can fight for 10+ minutes to multiple hours (Brownscombe *et al.,* 2017b). Here, we did not explore the effects of fight time on brook trout due to brief fight durations and limited variability in this factor, but fish size was still an important factor in brook trout reflex impairment, likely due to the greater effects of air exposure on larger individuals that have greater metabolic needs.

There is a comprehensive body of literature on brook trout thermal ecology and physiology, which was reviewed by Smith (2017). The average preferred temperature by brook trout in the laboratory was 15°C (range = 14.3–15.7 ± SE 0.3°C), but nine estimates of brook trout temperature selection in the wild found an average thermal preference of 13.7°C (range = 12.1–15.3°C ± SE 0.69), with maximum temperature values occupied at ~ 20–21°C (Wehrly *et al.,* 2007; Robinson *et al.,* 2010; Cote *et al.,* 2019). Based on laboratory studies that evaluated thermal response metrics including maximal growth, scope for activity and swimming performance, the brook trout fundamental thermal niche was estimated at 13–17°C (Smith, 2017). Our study documented greater brook trout reflex impairment and longer equilibrium recovery times at temperatures >19.5°C, which is fairly consistent with their thermal ecology and physiology. During warm surface-water periods, when brook trout may be residing in colder water at greater depths, it is likely especially important to minimize handling times and release fish quickly to allow them to return to colder water for recovery. The additional finding of increased loss of equilibrium due to longer air exposure times at >13.5°C is more surprising, as this is very close to their thermal preference and physiological optima. This may indicate that metabolic optima metrics (e.g. aerobic scope, scope for growth) are less relevant to physiological and behavioural responses to stressors such as air exposure than is metabolic rate, as it dictates oxygen needs, which increase near exponentially with temperature and body size (Clarke and Johnston, 1999). This is consistent with the hypothesis that metabolic needs or performance may be more relevant to fish fitness depending on the ecological scenario or stressor (Brownscombe *et al.,* 2017a).

In conclusion, there were strong interactive effects of air exposure duration, water temperature and fish size on reflex impairment in brook trout exposed to real or simulated recreational angling stressors. The simulated angling experiment resulted in greater reflex impairment responses than real angling, and hence, the thresholds developed here using both experiment types combined are likely conservative for real angling scenarios. These thresholds included greater impairment at air exposure durations >30 sec, and within these longer air exposure times, water temperature had a synergistic effect, exacerbating RAMP scores and time to equilibrium gain at >19.5°C and >13.5°C for equilibrium loss. Further, larger fish (>328 mm) were more vulnerable to equilibrium loss at >10 sec of air exposure. These findings are consistent with knowledge of fundamental biological processes and the C&R literature, which suggest synergistic effects result in negative fish health and survival impacts with longer air exposure, warmer temperatures and larger fish. The thresholds developed here provide a nuanced understanding of these interactions for C&R, as well as other scenarios where fish are being stressed (e.g. scientific sampling or experiments). Yet, it is important to note that there is certainly interspecies, and to some extent interpopulation, variation in these thresholds among these factors. The approach used here, with large sample sizes across a range of relevant factors and application of CIT as an analytical technique, may be of broad use to understanding this variation. As a general rule, reducing air exposure to <10 sec and avoiding upper thermal extremes for a given ecosystem remain well-supported approaches to achieve sustainable C&R fisheries. A general guideline such as the ‘10-sec rule’ will also remain easier to communicate and adopt by anglers and regulators, rather than a more nuanced approach that may create complexity and confusion about when the best practice matters. Nevertheless, identifying biologically relevant thresholds for C&R could help to further clarify and refine science-based guidance for regulators and anglers, thus improving outcomes for fish that are angled and released.

## Funding

This work was supported by an NSERC Engage Grant (#479161), in partnership with Kenauk Nature and the Kenauk Institute. S.C. is supported by NSERC and the Canada Research Chairs program. A.D. is supported by the National Institute of Food & Agriculture, U.S. Department of Agriculture, the Massachusetts Agricultural Experiment Station and Department of Environmental Conservation.
